# Frequent loss of RUNX3 gene expression in remnant stomach cancer and adjacent mucosa with special reference to topography

**DOI:** 10.1038/sj.bjc.6602372

**Published:** 2005-02-01

**Authors:** Y Nakase, C Sakakura, K Miyagawa, S Kin, K Fukuda, A Yanagisawa, K Koide, N Morofuji, Y Hosokawa, K Shimomura, K Katsura, A Hagiwara, H Yamagishi, K Ito, Y Ito

**Affiliations:** 1 Department of Surgery and Physiology of Digestive System, Graduate School of Medical Science, Surgery and Regenerative Medicine, Kyoto, Japan; 2 Department of Pathology, Graduate School of Medical Science, Surgery and Regenerative Medicine, Kyoto Prefectural University of Medicine, Kyoto, Japan; 3 Department of Surgery , Kyoto First Red Cross Hospital, Kyoto, Japan; 4 Department of Pathology, Kyoto First Red Cross Hospital, Kyoto, Japan; 5 Department of Surgery, Kyoto Second Red Cross Hospital, Kyoto, Japan; 6 Department of Pathology, Kyoto Second Red Cross Hospital, Kyoto, Japan; 7 Institute of Molecular and Cell Biology and Oncology Research Institute, National University of Singapore, 30 Medical Drive, Singapore 117609, Singapore

**Keywords:** RUNX3, carcinogenesis, remnant stomach cancer

## Abstract

Our previous studies suggest that a lack of RUNX3 function is causally related to the genesis and progression of human gastric cancer. This study was conducted to determine whether alteration of RUNX3 gene expression could be detected in the normal-looking gastric remnant mucosa, and to ascertain any difference in the potential of gastric carcinogenesis between the anastomotic site and other areas in the remnant stomach after distal gastrectomy for peptic ulcer (RB group) or gastric cancer (RM group), by analysing RUNX3 expression with special reference to topography. A total of 89 patients underwent distal gastrectomy for gastric cancer from the intact stomach (GCI group) and 58 patients underwent resection of the remnant stomach for gastric cancer (RB group: 34 cases, RM group: 24 cases). We detected RUNX3 and gene promoter methylation by *in situ* hybridisation, quantitative reverse transcriptase–polymerase chain reaction (RT–PCR), and methylation-specific PCR. The interval between the initial surgery and surgery for remnant gastric cancer (interval time) was 10.4 years in the RM group, and 27.5 years in the RB group. Cancers in the RB group were significantly more predominant in the anastomosis area (*P*<0.05). Within the tumour, downregulation of RUNX3 expression ranged from 74.7 to 85.7% in the three groups. The rate of downregulation of RUNX3 of adjacent mucosa was 39.2% (11 in 28 cases) in RB and 47.6% (10 in 21 cases) in RM, which are significantly higher than that of the GCI group (19.5%, 17 in 87 cases). In noncancerous mucosa of the remnant stomach in the RB group, RUNX3 expression decreased more near the anastomosis area. In the RM group, however, there were no significant differences in RUNX3 expression by sampling location. Based on RUNX3 downregulation and clinical features, residual stomach mucosa of the RM group would have a higher potential of gastric carcinogenesis compared to the RB or GCI group. Gastric stump mucosa of the RB group has higher potential especially than other areas of residual stomach mucosa. Measurement of RUNX3 expression and detection of RUNX3 methylation in remnant gastric mucosa may estimate the forward risk of carcinogenesis in the remnant stomach.

Our previous studies suggest that lack of RUNX3 function is causally related to the genesis and progression of human gastric cancer, indicating that RUNX3 is a novel tumour suppressor (Li *et al*, 2002). RUNX3 shows remarkable downregulation in gastric cancers compared to the surrounding mucosa, and the percentage of downregulation increases as the cancer stage progresses. Furthermore, RUNX3 expression is reduced in intestinal metaplasia, a precancerous state, compared with normal mucosa (Li *et al*, 2002), suggesting that downregulation of RUNX3 occurs at the early stages of gastric carcinogenesis, and that loss of RUNX3 expression increases the potential for gastric carcinogenesis.

Patients with a remnant stomach have been thought to be more susceptible to the development of cancer than the general population, perhaps related to duodenogastric reflux, denervation, and *Helicobacter pylori* infection (Toftgaard, 1989; Kaminishi *et al*, 1995; Miwa *et al*, 1993, 1995; Johannesson *et al*, 2003). In the long term after partial gastrectomy, atrophic gastritis, intestinal metaplasia, and dysplasia are frequent, and these changes are thought to be precancerous (Nakanishi *et al*, 1993; Safatle-Ribeiro *et al*, 1996; Tanigawa *et al*, 2000; Nishizawa *et al*, 2002). There are clinicopathologic differences between remnant gastric cancers arising after distal gastrectomy for peptic ulcer (RB group) *vs* gastric cancer (RM group). We hypothesised that the pathogenesis of the RM group, including its genetic background, differs from the RB group, but few studies have investigated these issues (Fujihara *et al*, 1996; Baba *et al*, 2001; Matsui *et al*, 2001).

The current investigation was undertaken to determine whether alterations of RUNX3 gene expression could be detected in normal-looking gastric remnant mucosa. We ascertained differences in the potential of gastric carcinogenesis between the anastomotic site and other areas in the remnant stomach after distal gastrectomy for peptic ulcer (RB group) or gastric cancer (RM group), by analysing RUNX3 with special reference to topography.

## MATERIALS AND METHODS

### Patients

We enrolled 89 patients with gastric cancer in the intact stomach (GCI group), and 58 patients with remnant gastric cancer after distal gastrectomy (RGC group). All GCI patients underwent distal gastrectomy for gastric cancer from the intact stomach between January 2001 and January 2002 at the Department of Digestive Surgery, Kyoto Prefectural University of Medicine. The RGC patients underwent resection of the remnant stomach for gastric cancer between January 1993 and September 2003 at the Department of Digestive Surgery, Kyoto Prefectural University of Medicine, the Department of Surgery, Kyoto First Red Cross Hospital, and the Department of Surgery, Kyoto Second Red Cross Hospital. The RGC group was divided into the RB group, 34 patients who initially underwent distal gastrectomy for benign peptic ulcer, and the RM group, 24 patients who underwent distal gastrectomy for primary gastric cancer. The Japanese General Rules for Gastric Cancer were used for pathologic diagnosis and classification of tumours (Japanese Gastric Cancer Association, 1999). The following factors were compared between groups: age, gender, depth of invasion, histologic type, lymph node spread, and stage. The following factors were compared between the RB and RM groups: interval between initial surgery and surgery for remnant gastric cancer (interval time), method of reconstruction after primary resection, and tumour location.

For the present study, we selected remnant gastric cancer cases in which cancers developed 5 or more years after initial distal gastrectomy for either peptic ulcer or gastric cancer, to avoid recurrent cancer cases.

### *In situ* hybridisation

Formalin-fixed, paraffin-embedded tissue blocks were cut into 5-*μ*m-thick sections in RNase-free water and mounted on slides. *In situ* hybridisation was performed as described previously (Satake *et al*, 1995) using sense and antisense DIG-labelled probes consisting of RUNX3 nucleotides 550–848 (accession number Z35278).

RNA probe was synthesised with T7 RNA polymerase, using a digoxigenin (DIG) RNA Labeling Kit (Boehringer Mannheim). After proteinase K digestion (18 *μ*g ml^−1^), the sections were postfixed with 4% (w v^−1^) paraformaldehyde in phosphate-buffered saline for 10 min and treated with 0.1 M triethanolamine-HCl (pH 8.0) for 1 min. Following acetylation for 10 min, the sections were dehydrated, air-dried and then incubated overnight at 50°C in hybridisation buffer composed of 50% formamide, 10 mM Tris-HCl (pH 7.5), 1 mg ml^−1^ yeast tRNA (Sigma), 1 × Denhalt's solution (Sigma), 10% PEG6000, 600 mM NaCl, 0.25% SDS, 1 mM EDTA, and 0.2 *μ*g ml^−1^ probe. After hybridisation, the sections were washed at 45°C for 1 h in 50% formamide and 2 × SSC, and digested with 20 *μ*g ml^−1^ RNase (Sigma) in 10 mM Tris-HCl (pH 8.0) and 500 mM NaCl at 37°C for 30 min. Hybridised DIG-labelled probes were visualised with a Nucleic Acid Detection Kit (Boehringer Mannheim).

As RUNX3 expression was weak at the stem cell zone of normal gastric mucosa, stained cells represented about 50% of epithelial cells in normal mucosa, and expressed in surface mucous cells and chief cells (Li *et al*, 2002). In the present study, we counted 1000 cells of the tumour or adjacent noncancerous epithelial cells to calculate the percentage of stained cells. We have counted the RUNX3-positive cells per gland base. RUNX3-positive cells in normal gland of different five areas were calculated and averaged. When it was not possible to count RUNX3-positive cells per gland base, we counted cells of the whole mucosal layer in each area. After counting more than 1000 epithelial cells, we calculated the percentage of positive cells, using all data from the three different zones. In the remnant mucosa, a percentage of RUNX3-positive cells less than 30% of that of the fundic gland was accepted as the criterion for downregulation of RUNX3 in remnant gastric mucosa.

### Quantitative reverse transcriptase–polymerase chain reaction

cDNA was produced from total RNA by using a Superscript preamplification system (BRL, Bethesda, MD, USA) and following the procedures suggested by the manufacturer. RNA was heated to 70°C for 10 min in 14 *μ*l of duethylpyrocarbonate-treated water containing 0.5 *μ*g oligo (dT). Synthesis buffer (10 ×), 2 *μ*l 10 mM dNTP mix, 2 *μ*l 0.1 M DTT, and reverse transcriptase (Superscript RT; 200 U *μ*l^−1^) were added to the sample. The resulting reaction mixture was incubated at 42°C for 50 min, and the reaction was terminated by incubating the mixture at 90°C for 5 min.

Quantitative PCR was performed using real-time ‘Taqman TM’ technology and analysed on a Model 5700 Sequence Detector (Applied Biosystems Corp., Foster City, CA, USA) as described previously (Sakakura *et al*, 2004).

RUNX3 reverse transcriptase-polymerase chain reaction (RT–PCR) primers are 5′-AAG CAC AGC CAT CAG GAT TCA-3′ and 5′-TGG ACA TGC TTG CGG ATA TAA G-3′. Hybridization probes, which bind to PCR products, were labelled with a reporter dye, FAM on the 5′ nucleotide and a quenching dye TAMRA, on the 3′ nucleotide. The sequence of hybridisation probe is RUNX3: 5′-(FAM) CAT CTG GAA CTT CTC CTG GTC TCT CAG C (TAMRA)-3′.

In total, 50 *μ*l reactions contained: 1.25 U Amp-*Taq* DNA polymerase, 1 × PCR reaction buffer, 180 ng of each primer, 200 *μ*M dNTP, 400 mM dNTP, 100 nM Taqman probe, and 0.5 U Amplirase (Applied Biosystems Corp.). The *C*_t_ value corresponding to the cycle number at which the fluorescence emission monitored in real time reaches a threshold of 10 standard deviations above the mean base line emission from cycle 1 to 40 was measured in serial dilutions of control cDNA, analysed for each target. These target genes were served as standard curves from which to determine the rate of changes of Ct value. Cycling parameters were 2 min at 50°C, and 10 min at 95°C followed by 40 cycles of 15 s at 95°C and 1 min at 60°C.

To minimise the errors arising from the varidation in the amount of starting RNA among samples, amplification of *β*-actin mRNA was performed as an internal reference against which other RNA values could be normalised. Normalised results were expressed as the ratio of copies of each gene to copies of the *β*-actin gene.

### Methylation-specific PCR

Methylation-specific PCR (MSP) was performed as reported previously (Herman *et al*, 1993). Briefly, genomic DNA denatured by NaOH was treated with sodium bisulphite and purified. The DNA was subjected to PCR using the following primers: primer set for untreated DNA: 5′-GAG GGG CGG CCG CAC GCG GG-3′, 5′-CGG CCG GCG CGG GCG CCT CC-3′; primer set for detecting methylated DNA: 5′-TTA CGA GGG GCG GTC GTA CGC GGG-3′, 5′-AAA ACG ACC GAC GCG AAC GCC TCC-3′; and primer set for detecting unmethylated DNA: 5′-TTA TGA GGG GTG GTT GTA TGT GGG-3′, 5′-AAA ACA ACC AAC ACA AAC ACC TCC-3′. Methylated nucleotides were verified by sequencing the PCR product.

### Statistical analysis

The results are presented as mean±s.d. values. Statistical comparisons between groups were performed using the Kruskal–Wallis test, the Mann–Whitney *U*-test, or Student's *t*-test by computer software StatMate (ATMS, Tokyo, Japan). Statistical significance was defined as a *P*-value less than 0.05.

## RESULTS

### Clinicopathologic variables

The clinicopathologic characteristics of patients in the GCI, RB, and RM groups are listed in Table 1. Age, gender, histologic type, and lymph node metastasis showed no significant differences between the three groups. The RB and RM groups had significantly more invasive tumours and more advanced stage cancers than the GCI group. Interval time and reconstruction at primary surgery and tumour location of the RB and RM groups are listed in Table 2. The interval time between primary surgery and later appearance of cancer was 27.5 years for RB and 10.4 years for RM. At the initial operation in the RB group, Billroth-II reconstructions were significantly more common than Billroth-I. By contrast, RM group patients had more Billroth-I reconstructions. In the RB group, remnant gastric cancers developed predominantly in the anastomosis areas and suture lines (25 in 34 cases, 73.5%). In contrast, in the RM group, there were no significant differences in tumour location.

### RUNX3 downregulation in remnant stomach cancers and surrounding mucosa analysed by *in situ* hybridisation

**Figure 1:**
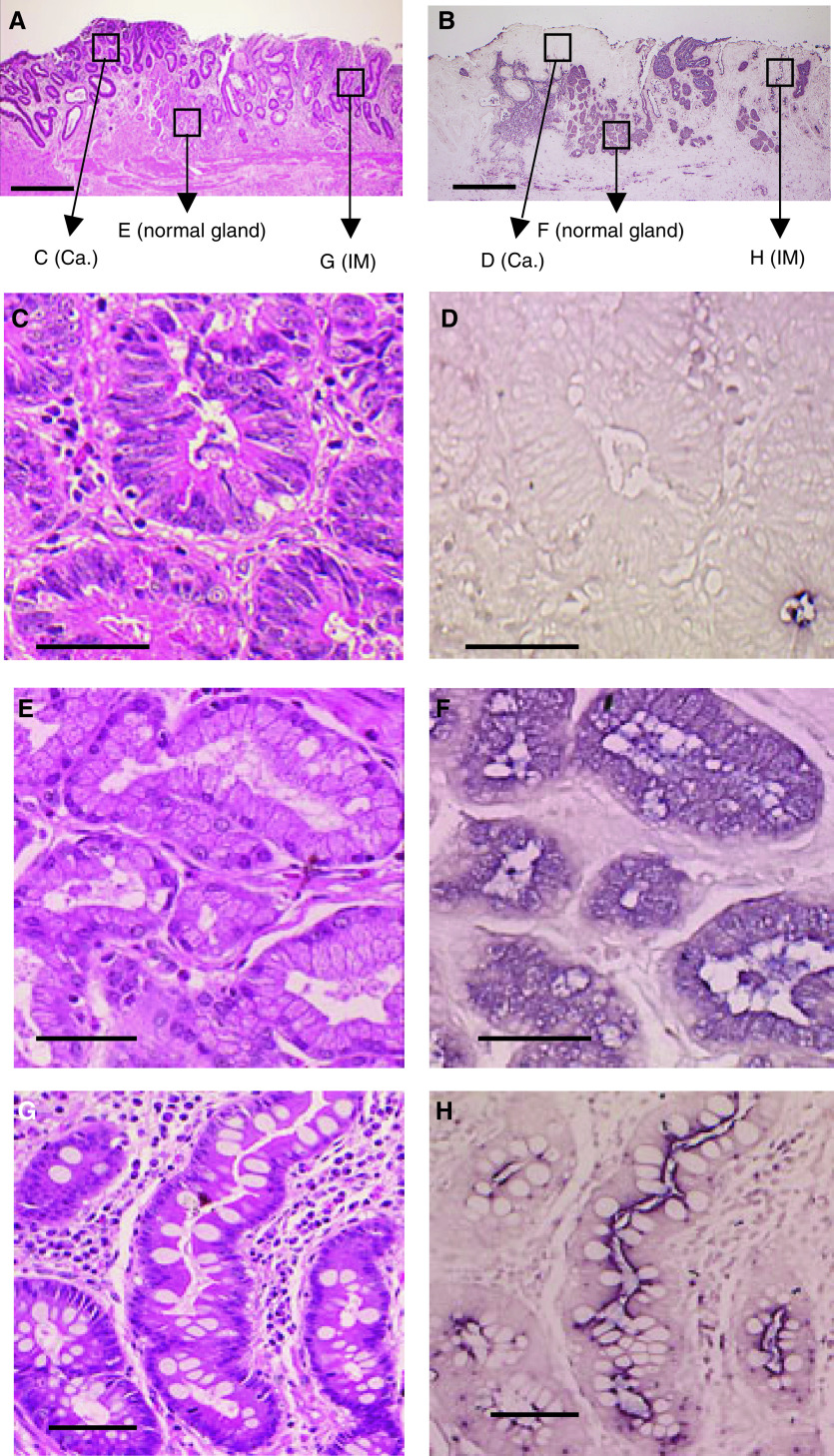
*In situ* hybridisation of RUNX3 mRNA in a remnant gastric cancer specimen. Haematoxylin and eosin staining (**A**) and *in situ* hybridisation using an antisense probe (**B**). Cancer tissue faces the lumen (upper side). Scale bars are equal to 1 mm. Enlargement of boxed regions marked (C–H) in (**A**) and (**B**). (**C**, **D**) show cancer cells (Ca.); (**E** and **F**) show normal mucosa; and (**G** and **H**) show intestinal metaplasia (IM). Scale bars are 100 *μ*m.

Figure 1 shows *in situ* hybridisation of RUNX3 mRNA in a remnant stomach cancer specimen. Panels A and B of Figure 1 show a surgically resected specimen of remnant stomach cancer tissue surrounded by normal mucosa of lower magnification (× 40). RUNX3 expression can be clearly detected within the normal gastric epithelial cell layer, but only weakly in the cancer tissue (Figure 1A and B). The pattern is more clearly visible at higher magnification. The corresponding regions in Figure 1C, E, and G are shown in Figure 1D, F, and H, clearly demonstrating that RUNX3 expression is mainly restricted to the normal epithelial cells and not evident in the surrounding mesenchymal cells (× 400). Figure 1C shows cancer cells of well-differentiated adenocarcinoma with irregularly shaped atypical tubular structures lined by pleomorphic cells and enlarged, irregular, vesicular nuclei. Figure 1D demonstrates that cancer tissue does not significantly express RUNX3. In contrast, intensive RUNX3 expression is observed in Figure 1E and F. Intestinal metaplasia shows downregulation of RUNX3 (Figure 1G and H). The result of a negative control is shown when the antisense probe is replaced by a sense probe (data not shown). Our additional experiments have shown that *in situ* hybridisation with *β*-actin as a positive control shows the positive staining of the whole area of mucosa (data not shown).

**Figure 2:**
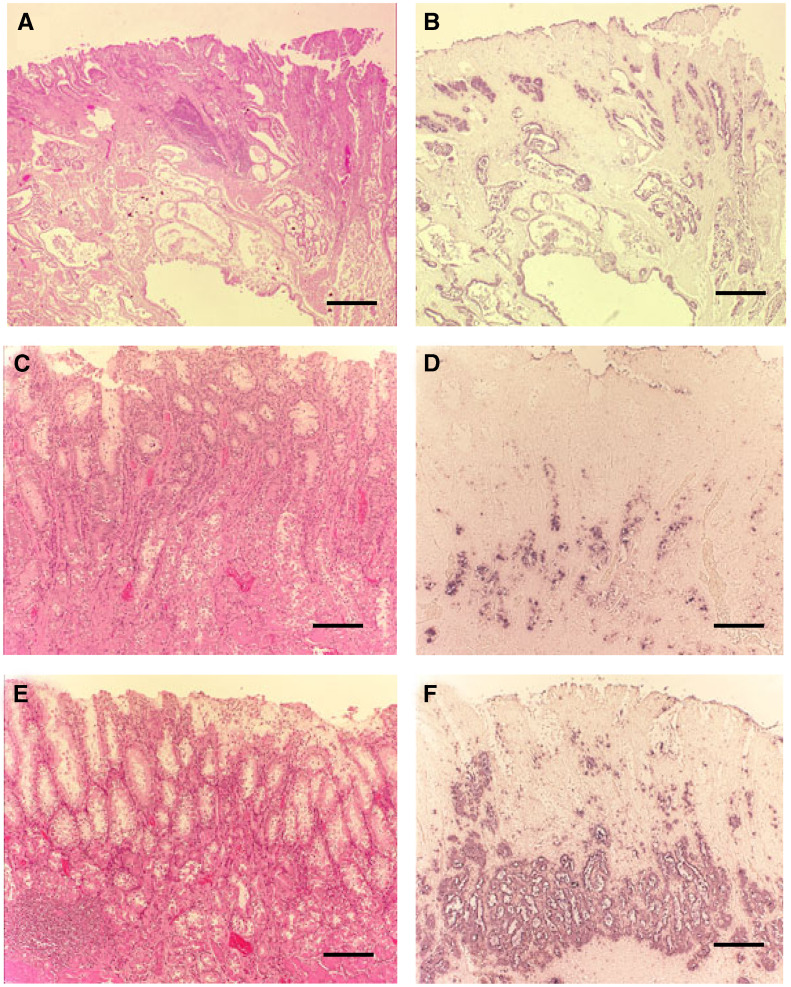
RUNX3 expression in noncancerous remnant gastric mucosa (RB group, Billroth-II reconstruction). (**A**, **B**) are gastric cystica polyposa in the anastomosis area, (**C**, **D**) are distal, and (**E**, **F**) are proximal. Haematoxylin and eosin staining (**A**, **C**, **E**) and *in situ* hybridisation using antisense probe (**B**, **D**, **F**). Mucosa faces the lumen (upper side). Scale bars are 300 *μ*m.

Gastric stump mucosa of the RB group showed significant downregulation of RUNX3 compared to other areas (distant areas from gastric stump) as shown in Figure 2. Furthermore, gastric cystica polyposa (GCP) was frequently observed in gastric stump mucosa and typical data of RUNX3 expression in these lesions were analysed by *in situ* hybridization as shown in Figure 2. Figure 2B shows significant downregulation of RUNX3 in GCP, and HE staining of corresponding lesions is shown in Figure 2A. In both intestinal metaplasia and chronic gastritis, RUNX3 expression was reduced. RUNX3 expression was decreased to a greater extent near the anastomosis area in the RB group (Figure 2C and D). In contrast, RUNX3 expression was observed mainly distant from the anastomosis area in the RB group (Figure 2E and F).

### Incidence of RUNX3 downregulation in remnant stomach cancers and surrounding mucosa

**Figure 3:**
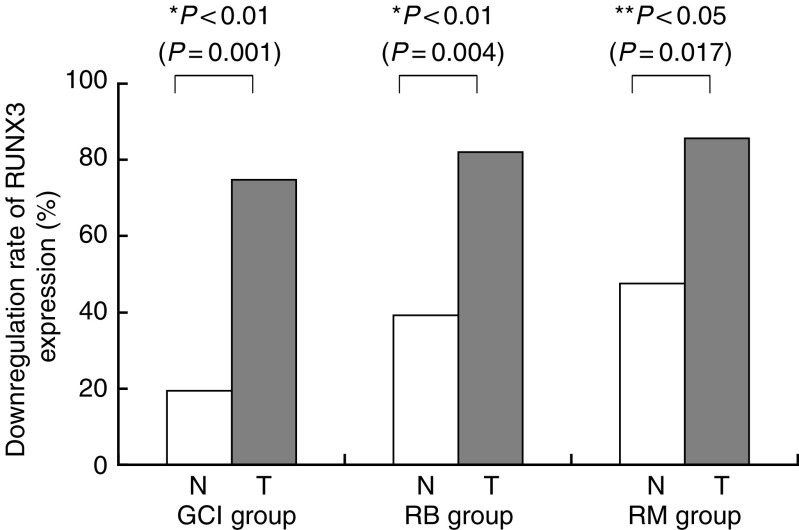
Downregulation rate of RUNX3 expression in mucosa and tumour in GCI, RB, and RM groups. T; tumour. N; noncancerous adjacent mucosa.

Informative results were obtained in 87 of 89 GCI cases, 28 of 34 RB cases, and 21 of 24 RM cases. The overall incidence of RUNX3 downregulation in clinical specimens of RM, RB, and GCI cases is shown in Figure 3. Downregulation of the RUNX3 expression rate in tumour was 74.7% (65 in 87 cases) in the GCI group, 82.1% (24 in 28 cases) in the RB group, and 85.7% (18 in 21 cases) in the RM group. RUNX3 downregulation in cancerous tissues of each group (RM, RB, and GCI group) was frequently observed in the same degree (74.7, 82.1, and 85.7%, respectively). In contrast, the rate of downregulation of RUNX3 in adjacent mucosa was 39.2% (11 in 28 cases) in RB and 47.6% (10 in 21 cases) in RM, significantly higher than that of the GCI group (19.5%, 17 in 87 cases).

### RUNX3 downregulation in remnant stomach cancers and surrounding mucosa analysed by quantitative RT–PCR

**Figure 4:**
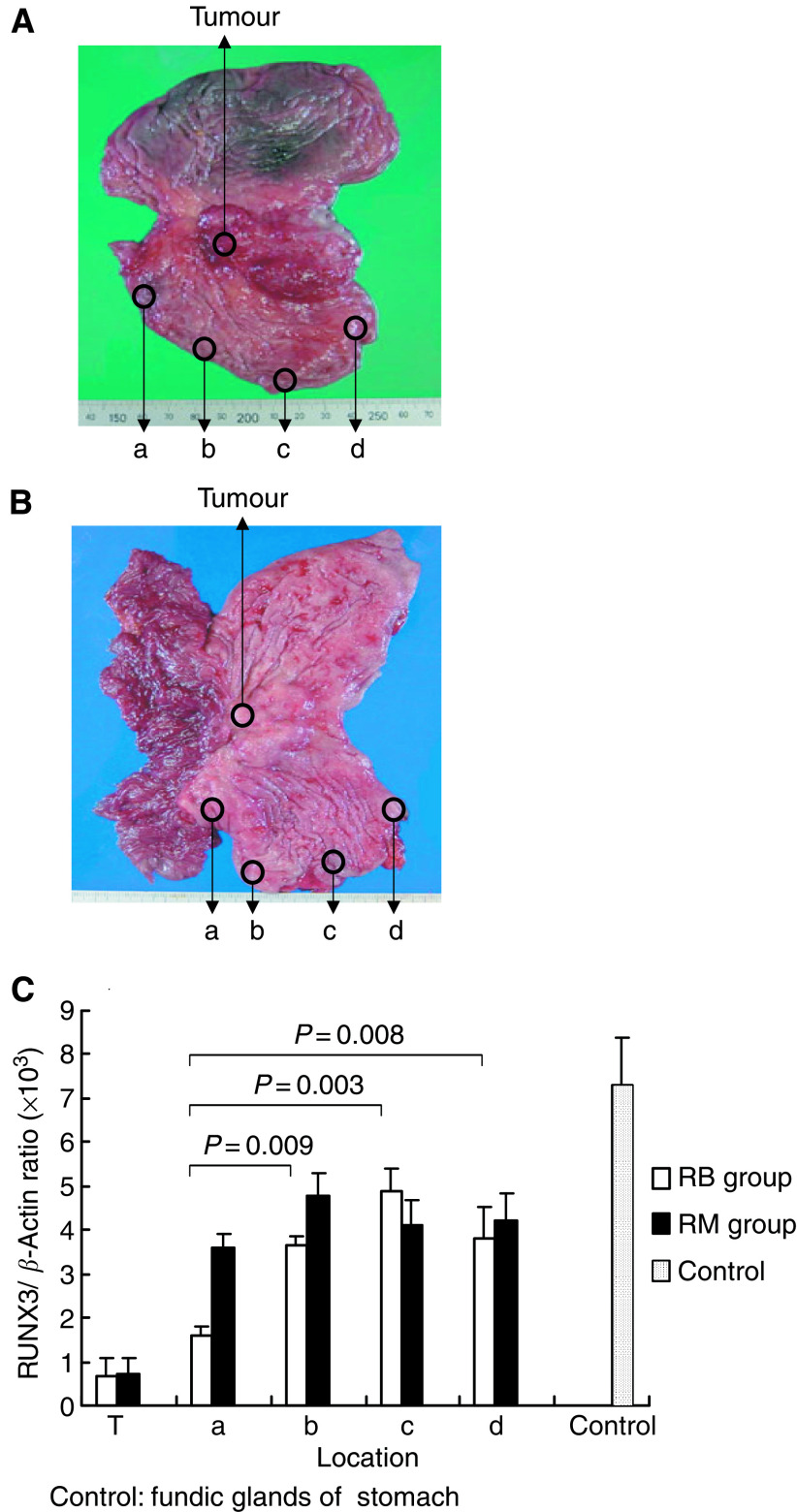
Quantitative RT–PCR of noncancerous mucosa in RB and RM groups. Resected remnant stomach; RM group, Billroth-I reconstruction (**A**). RB group, Billroth-II reconstruction (**B**). Gastric mucosa samples from remnant stomach are 2–3 cm lengths from distal to proximal (a–d). (**C**) Quantitative analysis of tumour and noncancerous mucosa (a–d) in RM (black bars) and RB groups (white bars). Control; normal gastric mucosa (fundic glands). Mean±s.d. values.

Gastric mucosa samples were taken from the resected remnant stomach at 2 cm lengths from distal to proximal as shown in Figure 4A and B. Figure 4A shows the surgical specimen of remnant stomach cancer arising after distal gastrectomy for gastric cancer reconstructed by Billroth-I procedure (RM group). Figure 4B shows the surgical specimen of remnant stomach cancer arising after distal gastrectomy for peptic ulcer reconstructed by Billroth-II procedure (RB group). We performed quantitative RT–PCR in 12 RB cases and 10 RM cases. We measured RUNX3 expression in 10 cases with normal mucosa as a reference, sampled from the intact stomach by endoscopy. As shown in Figure 4C, RUNX3 expression in all areas of remnant gastric mucosa in both RB and RM groups was significantly more decreased than in normal gastric mucosa (*P*<0.01). Furthermore, RUNX3 expression was significantly decreased near the anastomosis area (location a of panel B, Figure 4) in the RB group compared to other areas (locations b, c, d, and e of panel B, Figure 4) (Figure 4, panels B and C, *P*<0.05). In contrast, in the RM group, there were no significant differences in RUNX3 expression in the surrounding mucosa for all sampling locations (Figure 4, panels A and C, locations a, b, c, and d).

### Methylation-specific PCR

**Figure 5:**
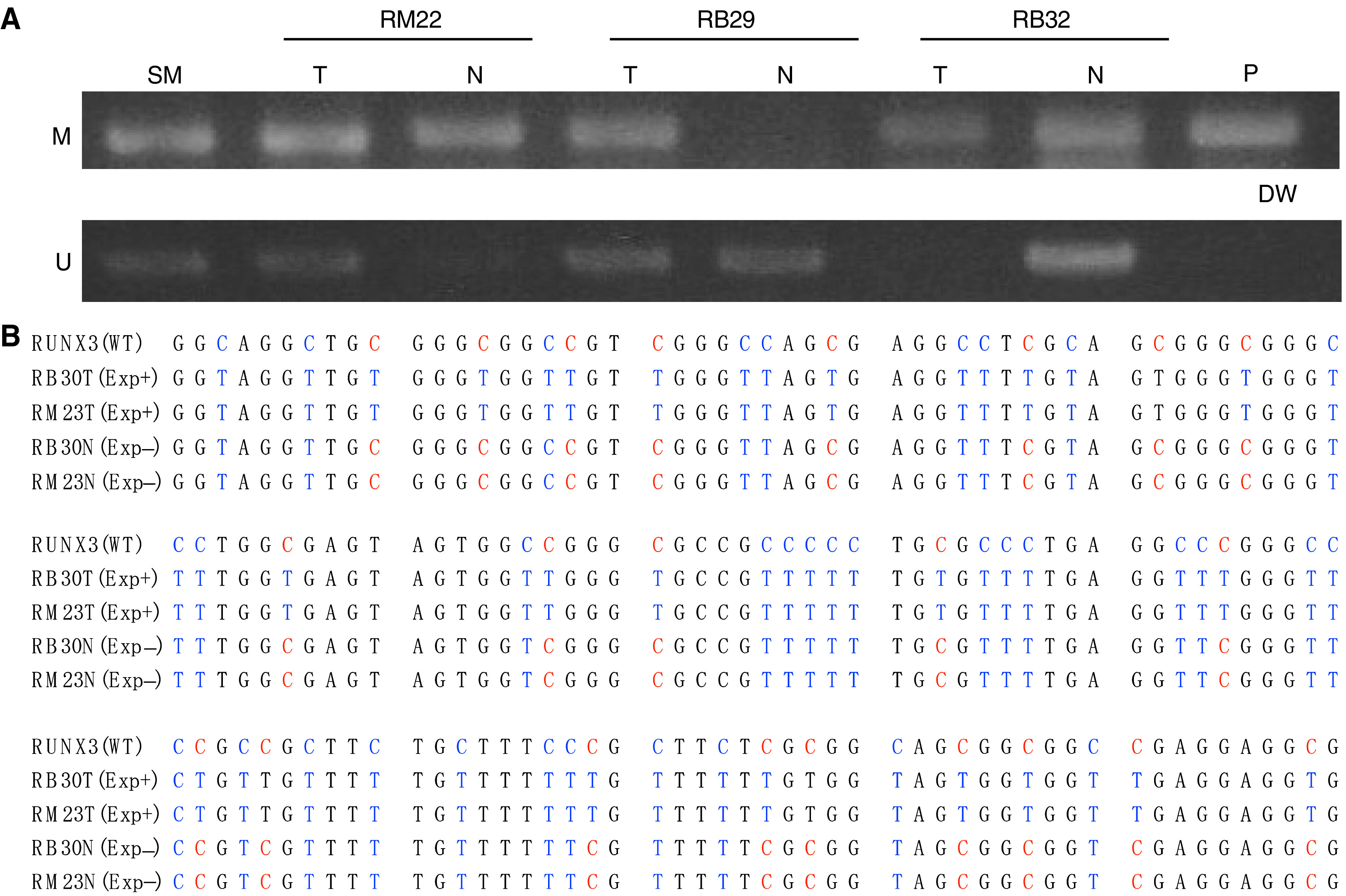
(**A**) Methylation-specific PCR of remnant gastric cancer and adjacent mucosa. (M) Methylated-sequence-specific PCR. (U) Unmethylated-sequence-specific PCR. Methylated PCR product present in T and N of RM22 and RB32, and T in RB29, unmethylated PCR products in T and N of RB29, T in RM22, and N in RB32. Lanes: T, tumour; N, noncancerous mucosa; P, positive control; DW, distilled water; and SM, size marker. (**B**) Methylation status of the C residues between −218 and −69 relative to the translation initiation site of the RUNX3 exon 1 region. The nucleotide sequences of the products of MSP of the DNA samples from remnant gastric cancer that do not express RUNX3 (Exp−) (RB30T, RM23T) and noncancerous mucosa that express RUNX3 (Exp+) (RB30N, RM23N) together with wt RUNX3 sequence are at the top. The red C indicates that it was resistant to bisulphite treatment due to methylation. The blue T indicates that it was converted from C by bisulphite treatment, that is, not methylated.

We performed MSP in 12 RB cases and 10 RM cases. Typical data for MSP of remnant gastric cancer and adjacent mucosa are shown in Figure 5A ((M) methylated-sequence-specific PCR, (U) unmethylated-sequence-specific PCR). Methylation of RUNX3 was present in 63.6% (14 in 22 cases) of cancers and 27.2% (six in 22 cases) of noncancerous adjacent mucosa, which correlated with RUNX3 expression. A significant correlation between methylation status of RUNX3 and RUNX3 expression was observed. In samples that were negative for RUNX3 expression, C residues in the CpG dinucleotide within the region were methylated (Figure 5B). In contrast, in samples positive for RUNX3 expression, none of the C residues were methylated.

## DISCUSSION

In the present study, we noted two significant differential features between RM and RB groups: tumour location and interval time. Cancers in the RB group were significantly more predominant in the anastomosis area, and the interval time in the RM group was shorter. These clinicopathologic features suggest that residual stomach mucosa of RM would have higher potential for gastric carcinogenesis compared to the RB and GCI groups and that gastric stump mucosa of RB has a higher potential than other parts of the residual stomach mucosa.

In accordance with clinicopathologic analysis, significant downregulation of RUNX3 was observed in the whole mucosa of the RM group and the anastomotic site of the RB group. In the remnant stomach mucosa, chronic gastritis and intestinal metaplasia were intensively observed with downregulation of RUNX3. Our previous study and others indicate that RUNX3 downregulation is frequently observed in chronic gastritis, intestinal metaplasia, and gastric adenoma in the GCI group (Li *et al*, 2002; Kim *et al*, 2004), which are thought to be precancerous (Safatle-Ribeiro *et al*, 1996; Tanigawa *et al*, 2000; Nishizawa *et al*, 2002). In our study, 19.5% of adjacent mucosa of the GCI group had downregulation of RUNX3 expression. Although there is no downregulation of RUNX3 in gastric mucosa at the initial operation for benign peptic ulcer (data not shown), the downregulation of RUNX3 in noncancerous mucosa has already occurred by the time of primary surgery in the RM group (19.5%), suggesting that the background mucosa of RM has a high risk of carcinogenesis at the time of primary surgery. In cases of remnant gastric cancer, the rate of downregulation of RUNX3 of adjacent mucosa was 39.2% in RB and 47.6% in RM, which are higher than that of GCI (19.5%). Based on the incidence of RUNX3 downregulation in background gastric mucosa, it is suggested that the risk of carcinogenesis in the RB or RM groups is higher than that of the GCI group. RUNX3 downregulation would have a causal role in carcinogenesis of the remnant stomach, as well as GCI.

An increased risk of remnant gastric cancer at the anastomotic site has been reported (Miwa *et al*, 1995). ‘Gastric stump carcinoma’ is primarily recognized as a carcinoma developing in the gastric remnant after gastrectomy for benign disease (Freedman and Berne, 1954; Heinzel and Laqua, 1954). The stump carcinoma was often localised to the anastomosis, known to be the site of severe duodenogastric reflux. For this reason, the argument has been made about the role of duodenogastric reflux as an important causal factor in gastric carcinogenesis. Previous studies indicate that patients with Billroth-II reconstruction tend to have cancer development at the anastomotic site, while patients with Billroth-I reconstruction tend to have cancer development at nonanastomotic sites (Kondo *et al*, 1991; Sowa *et al*, 1993; Baba *et al*, 2001; Tanigawa *et al*, 2002). Our results were similar, but patients with Billroth-II reconstruction of the RM group had equivalent rates at both sites. In accordance with tumour location, RUNX3 expression was more decreased in gastric stump mucosa in the RB group, and there was no difference in RUNX3 expression by location in the RM group. Gastric cystica polyposa, which is suspected to be of great relevance with regard to cancer development in the remnant stomach, is detected more often at the anastomotic site of patients with Billroth-II reconstruction (Koga *et al*, 1979; Matsuda *et al*, 1995). Interestingly, RUNX3 is downregulated in GCP as shown in Figure 2, although it is still controversial whether GCP is precancerous or not (Littler and Gleibermann, 1972; Jablokow *et al*, 1982; Mukaisho *et al*, 2003). These findings suggest that methylation and silencing of RUNX3 occurs mostly at the anastomotic site in the RB group, probably due to bile reflux. The interval time was 27.5 years for the RB group and 10.4 years in the RM group, similar to previous reports (Koga *et al*, 1979; Fujihara *et al*, 1996; Baba *et al*, 2001). Carcinogenesis in RB through bile reflux, denervation, or *Helicobacter pylori* infection may take longer than re-emergence of cancer on RM.

To date, genetic alterations associated with remnant gastric cancer have included p53, Ki-ras, cyclooxygenase-2, and bcl-2 (Clarke *et al*, 1997; Yamashita *et al*, 1998; van Rees *et al*, 1999; Baba *et al*, 2001; Kawabe *et al*, 2002). However, no reports have addressed these issues through pathogenesis and genetic changes of the surrounding mucosa. Few studies have investigated the genetic differences between RB and RM groups. Baba *et al* (2001) compared these groups and reported that p53 mutations and PCNA overexpression enhanced the proliferative activity of cancer cells of the RM group (Fukuzawa *et al*, 1996). Many genetic changes, including RUNX3 downregulation, could contribute to the increased potential for gastric carcinogenesis. Further studies are necessary to clarify this.

In conclusion, based on RUNX3 downregulation and clinical features, residual stomach mucosa of the RM group has higher potential for gastric carcinogenesis compared to the RB or GCI groups. Gastric stump mucosa of the RB group has higher potential than other areas of residual stomach. Although most patients with remnant gastric cancer have advanced disease and a poor prognosis (Sakakura *et al*, 2004), endoscopy for monitoring the remnant stomach may contribute to early detection and improved prognosis (Kikuchi *et al*, 2003). The measurement of RUNX3 expression and detection of RUNX3 methylation in remnant gastric mucosa may estimate the forward risk of carcinogenesis in the remnant stomach.

## Figures and Tables

**Table 1:** Clinicopathologic characteristics of patients with gastric cancer from intact stomach (GCI group) and remnant gastric cancer (RB group and RM group)

	**GCI (89)**	**RB 34**	**RM 24**	***P*-value**
Age (years)	66.1±13.7	66.3±10.5	62.7±9.5	NS
				
Male : female ratio	62 : 27	30 : 4	19 : 5	NS
				
Depth of invasion**				
m	23	5	2	
sm	28	6	6	
pm	6	3	4	0.034
ss	8	7	4	
se	20	9	4	
si	4	4	4	
				
*Histologic type*				
tub 1	24	12	7	
tub 2	15	5	4	
por 1	12	4	3	
por 2	17	7	4	NS
sig	16	4	5	
pap	5	1	1	
				
*Lymph node spread*				
n0	55	22	17	
n1	18	4	3	NS
n2	10	4	2	
n3	6	2	2	
				
Stage classification*				
Ia	47	10	6	
Ib	13	4	4	
II	9	2	2	0.004
IIIa	7	7	5	
IIIb	7	5	2	
IV	6	4	5	

* *P*<0.01,

** *P*<0.05, NS: not significant. GCI=cancer from intact stomach. RB=remnant gastric cancer arising after distal gastrectomy for peptic ulcer. RM=remnant gastric cancer arising after distal gastrectomy for gastric cancer.

**Table 2:** Clinical characteristics of the RB and RM groups

	**RB (34)**	**RM (24)**	***P*-value**
Interval (years)*	27.5±10.5	10.4±4.1	0.001
			
*Reconstruction at primary surgery* *			
Billroth I : II	9 : 25	17 : 7	0.003
			
Location of tumour* (*Billroth I : II*)			
Anastomosis	19 (2 : 17)	6 (3 : 3)	
Suture-line	6 (3 : 3)	2 (2 : 0)	0.021
Other	6 (4 : 2)	15 (11 : 4)	
Wide body	3 (1 : 2)	1 (1 : 0)	

Interval=between primary surgery and the appearance of remnant gastric cancer.

* *P*<0.01, NS: not significant.
